# Congenital Pleomorphic Adenoma in a SubmandibularGland of a Newborn- A Case Report

**Published:** 2016-03

**Authors:** Roxana Azma, Minoo Fallahi, Maliheh Khoddami, Bibi Shahin Shamsian, Samin Alavi

**Affiliations:** 1 *Department of Pediatric Radiology, Mofid Children’s Hospital, Shahid Beheshti University of Medical Sciences, Tehran, Iran.*; 2 *Neonatal Health Research Center (NHRC), Shahid Beheshti University of Medical Sciences, Tehran, Iran.*; 3 *Pediatric Pathology Research Center, Mofid Children’s Hospital, Shahid Beheshti University of Medical Sciences, Tehran, Iran.*; 4 *Pediatric Congenital Hematologic Disorders Research Center, Mofid Children’s Hospital, Shahid Beheshti University of Medical Sciences, Tehran, Iran.*

**Keywords:** Newborn, Pleomorphic adenoma, Submandibular gland neoplasm

## Abstract

**Introduction:**

Pleomorphic adenoma is a rare benign salivary gland neoplasm in children, which can be treated by simple excision. This tumor is rarely included in the differential diagnosis of solid submandibular masses in children. In the neonates, congenital pleomorphic adenoma usually presents in the nasopharynx. Surgical excision is the treatment of choice and recurrence is not expected. We report what appears to be the first case of congenital pleomorphic adenoma in the submandibular region in a one-day-old newborn.

**Case Report:**

The case of a one-day-old term baby is presented with a 5x2 cm left submandibualr mass with extension to the oral cavity. The mass was hard and non-mobile. During Ultrasonography and Contrast-enhanced Computed Tomography (CT) scan, the mass was solid with a heterogeneous internal structure. The tumor was completely excised and proved to be a pleomorphic adenoma during histopathological examination.

**Conclusion:**

Congenital pleomorphic adenoma rarely occurs in the nasopharynx and is treated by surgical excision. Our case is unique because the congenital pleomorphic adenoma is located in the submandibular gland of a newborn.

## Introduction

Pleomorphic adenoma (mixed tumor) of the salivary gland, although a well-recognized tumor in adults, is a rare tumor in the pediatric population. These tumors usually occur in the parotid gland followed by the submandibular gland and are rarely included in the differential diagnosis of solid submandibular masses in children (1). In the neonates, congenital pleomorphic adenoma usually presents in the nasopharynx (2). This report presents what appears to be the only case of congenital pleomorphic adenoma arising in the submandibular gland of a neonate in the published English literature. 

## Case Report

A one-day old term newborn with a birth weight of 3500 gr who was delivered by cesarean, was referred to our hospital because of a large left submandibualr mass. Upon physical examination a 5x2 cm non-mobile hard mass with no overlying skin erythma was present in the left submandibular region with extension to the oral cavity. The baby was not ill or toxic. 

**Fig 1:**
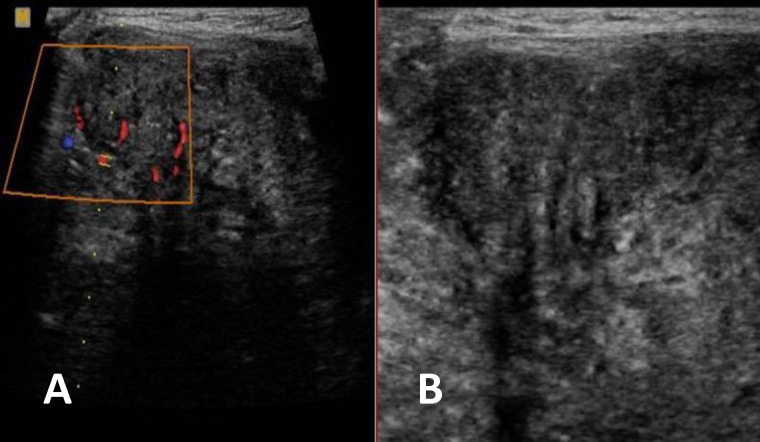
Cervical US scan in axial plane through the left Submandibular region (A,and B)shows a heteroechoic solid mass containing scattered hyperechoic foci(arrow) . Color Doppler imaging in the same axial scanning plane (A) shows vascularization within the mass

The moro and grasp reflexes were normal; however, the sucking reflex was impaired. Imaging was performed for the patient. Ultrasonography revealed a large hetero- echoic solid mass containing scattered hyperechoic foci and internal vascular flow (Fig.1). 

**Fig 2:**
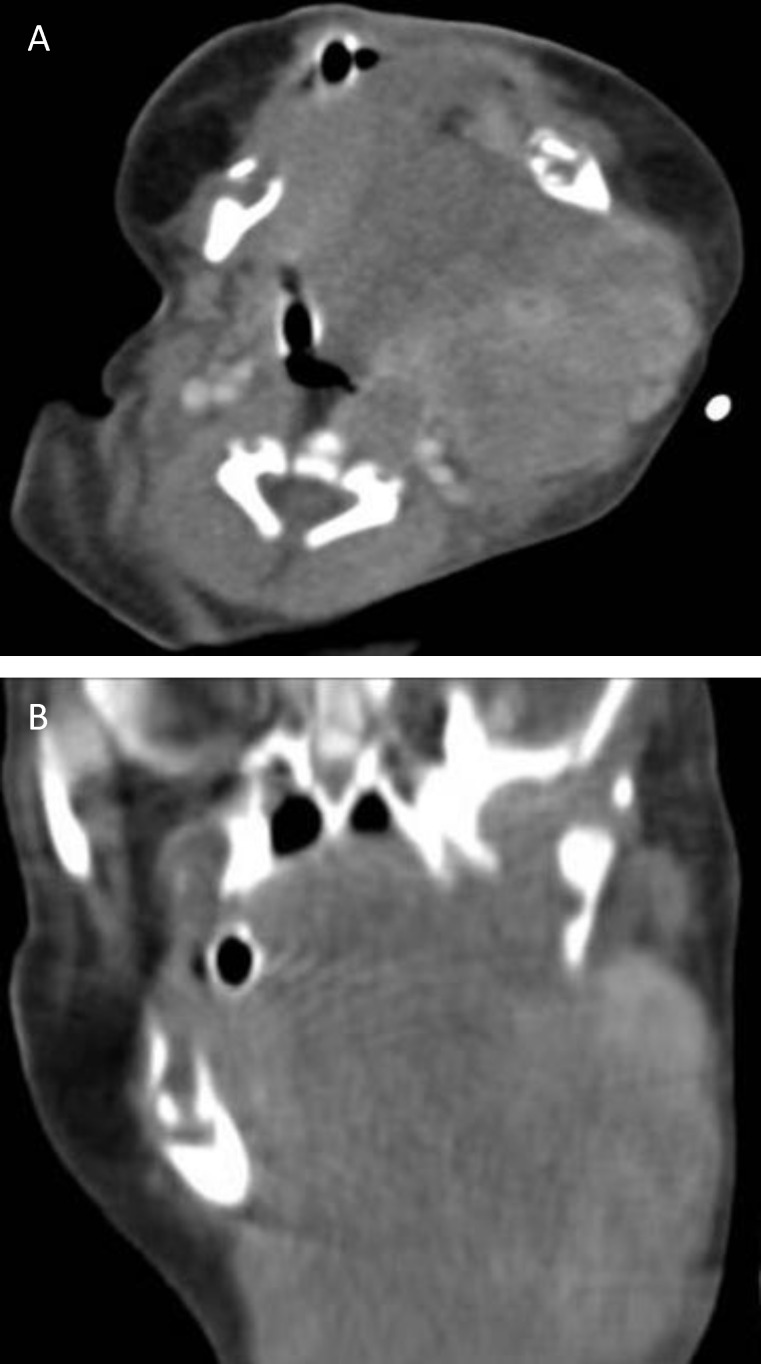
Contrast-enhanced CT in Axial 1,2 mm thickness images (A) and coronal 2mm thickness reconstruction images (B) demonstrate a large multicompartmental  hypodense lesion(arrows) with moderate heterogeneous enhancement in the  left submandibular space involving the sublingual space and oral cavity

Contrast-enhanced Computed Tomography (CT) scan showed a multicompartmental 5.7x5.7x6.5 cm hypodense lesion with moderate heterogeneous enhancement in the left submandibular area extending to the sublingual space and left half of the tongue through the posterior aspect of the left mandibular ramus (Figs. 2A, 2B).

The oropharyngeal cavity was almost obliterated by the lesion. Teratoma was suspected and serum levels of BHCG and alpha fetoprotein were checked which were within normal limits. Open biopsy was performed which revealed a pleomorphic adenoma characterized by a mixture of glandular epithelial/ myoepithelial compo- nent and fibromyxoid stroma containing areas of cartilaginous differentiation.

**Fig3:**
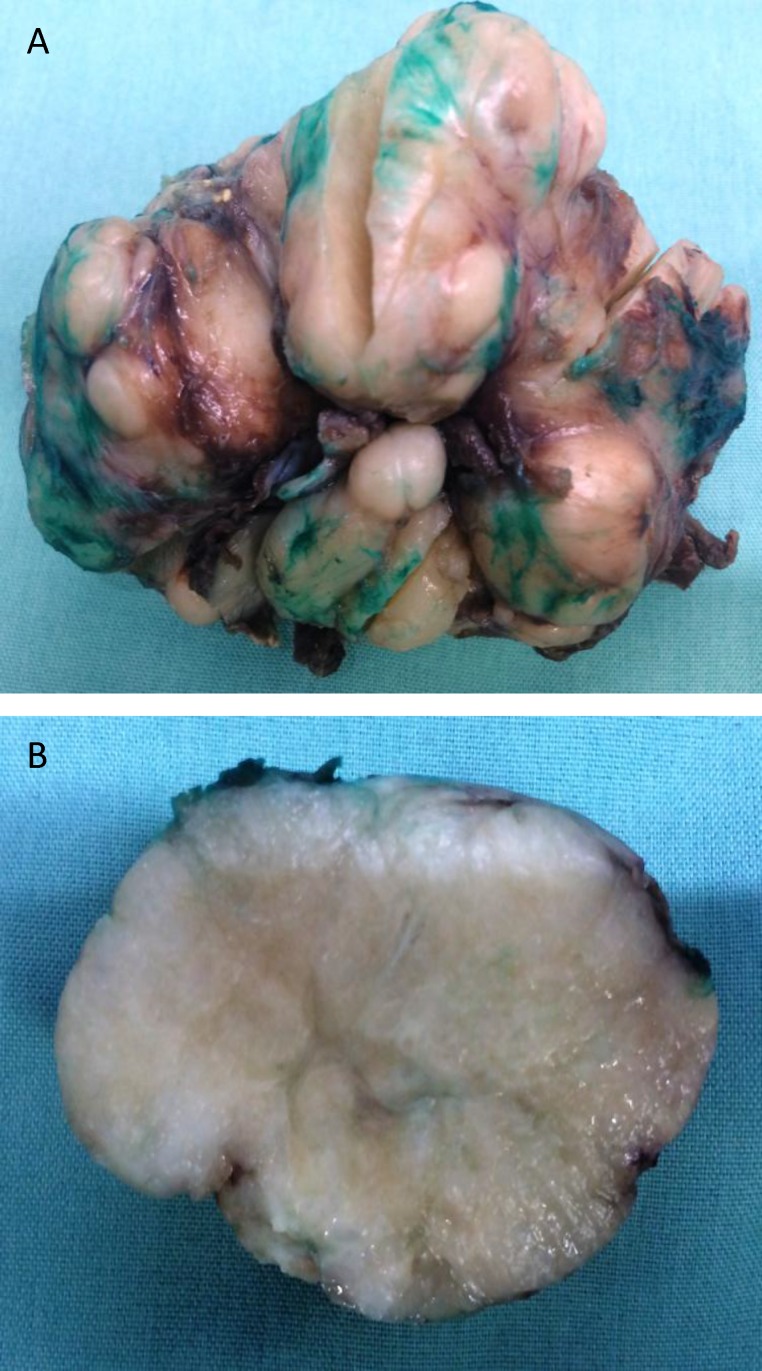
Macroscopic pathology: In gross examination of the surgical specimen, the resected piece was an encapsulated, bosselated tan firm tumor (A). Cut surface was tan-white and nodular with chondroid appearance (B

 The tumor was completely excised. It was an encapsulated, bosselated, **6.5x6x6 cm, and** firm with tan-white nodular chondroid cut surfaces (Figs. 3A,3B).

**Fig 4:**
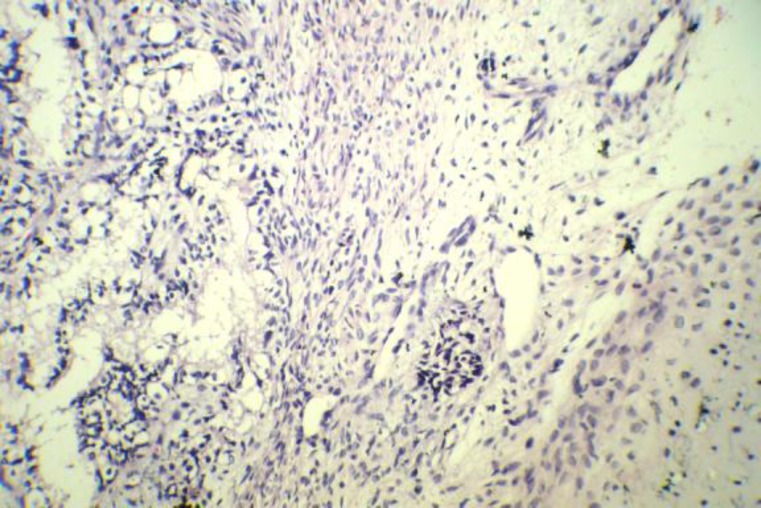
Microscopic pathology: Pathological study of the surgical specimen (hematoxylin and eosin staining, magnification: 250x) revealed admixture of glandular epithelial/myoepithelial component and fibromyxoid stroma with cartilaginous differentiation

On microscopic examination, features similar to the biopsy specimen were present with extensive chondroid differentiation (Fig.4). No evidence of malignancy was identified in the multiple sections that were examined. 

## Discussion

Most head and neck masses in children are benign. Congenital origin is suggested when a painless mass is present since birth or diagnosed soon after birth (3). The most common congenital neck masses in children are thyroglossal duct remnants followed by branchial cleft cysts (4).

Regarding the salivary glands, inflame- matory and vascular lesions are the most common ones. Teratomas and dermoid cysts which are relatively rare in the cervical region are true congenital neoplasms (5). Salivary gland tumors constitute only 1% of all head and neck neoplasms. Furthermore, less than 5% of the salivary gland tumors occur in children less than 16 years old (6).

Most of salivary gland neoplasms are benign. Pleomorphic adenoma, which is the most common benign salivary gland neoplasm, presents very rarely in children and most commonly affects the parotid gland. The involvement of the submandibular gland is extremely rare and among the submandibular triangle tumors, pleomorphic adenoma comprises merely 4% of cases in children (1,7).

In neonates, congenital pleomorphic adenoma, also known as salivary gland anlage tumor (SGAT), is a rare tumor that occurs in the nasopharynx (2). It is considered as a hamartoma of minor salivary glands and presents as a polypoid lesion in the nasopharynx, which leads to respiratory distress upon birth or in the early weeks of life (8). Several cases of this tumor have been reported in literature (2,8-15). 

Pleomorphic adenoma is a histologically diverse tumor, therefore noted to be a benign mixed tumor. They are derived from a mixture of ductal and myoepithelial cells and reveal both epithelial and mesenchymal differentiation. This tumor tends to be firm and mobile with no evidence of fixation to the mandible or the floor of oral cavity (16).

During ultrasonography, the tumor is either hypoechoic or isoechoic to the rest of the normal gland parenchyma with occasional hyperechoic calcified foci. CT scan or MRI features are commensurate with the size of the tumor, which is homogeneous and well-defined in small lesions and more heterogeneous and less well defined in larger ones (17). Imaging cannot differentiate between the benign and malignant types unless the tumor shows evidence of invasion to the surrounding tissues (16). The benign nature of the tumor can be confirmed by fine needle aspiration (FNA) or biopsy (17).

In our case, physical examination revealed a firm submandibualr mass, which was in favor for the solid nature of the lesion. Ultrasonogarphy and CT scan also confirmed the solid mass with internal vascular flow. Scattered hyperechoic foci were also present in the mass during ultrasonography. The extension of the mass to the oral cavity with no evidence of bony remodeling or discernible abnormality was shown in the CT scan. Consequently, the more common cystic congenital masses were ruled out and teratoma was our first diagnosis. However, open biopsy proved it to be a pleomorphic adenoma with no evidence of malignancy. This diagnosis was confirmed afterwards in the specimen from total surgical excision of the mass. Treatment of pleomorphic adenoma includes complete resection of the mass. which was performed for our patient with a satisfactory result. Rupture of the tumoral capsule or misunderstood tumoral margin can lead to local recurrence(1).

## Conclusion

Congenital pleomorphic adenoma rarely occurs in the nasopharynx, which is treated by surgical excision. Our case is unique because the congenital pleomorphic adenoma is located in the submandibular gland of a newborn. 
